# Methylation pattern and mRNA expression of synapse-relevant genes in the MAM model of schizophrenia in the time-course of adolescence

**DOI:** 10.1038/s41537-022-00319-8

**Published:** 2022-12-08

**Authors:** Abdul Qayyum Khan, Lukas Thielen, Gwenaëlle Le Pen, Marie-Odile Krebs, Oussama Kebir, Adrian Groh, Maximilian Deest, Stefan Bleich, Helge Frieling, Kirsten Jahn

**Affiliations:** 1 Laboratory for Molecular Neurosciences (LMN), Department of Psychiatry, Social Psychiatry and Psychotherapy, Medical School Hannover (MHH), Carl-Neuberg-Str. 1, 30625 Hannover, Germany; 2 University of Management and Technology—School of Pharmacy, 72-A Raiwind Rd, Dubai Town, Lahore Pakistan; 3 Université Paris Cité, Institute of Psychiatry and Neuroscience of Paris (IPNP), INSERM, Pathophysiology of Psychiatric disorders: Development and Vulnerability, U1266, 102-108 Rue de la Santé, 75014 Paris, France; 4 GHU Paris Psychiatrie et Neurosciences, 1 Rue Cabanis, 75014 Paris, France

**Keywords:** Epigenetics in the nervous system, Synaptic transmission, Molecular neuroscience

## Abstract

Schizophrenia is highly heritable and aggregating in families, but genetics alone does not exclusively explain the pathogenesis. Many risk factors, including childhood trauma, viral infections, migration, and the use of cannabis, are associated with schizophrenia. Adolescence seems to be the critical period where symptoms of the disease manifest. This work focuses on studying an epigenetic regulatory mechanism (the role of DNA methylation) and its interaction with mRNA expression during development, with a particular emphasis on adolescence. The presumptions regarding the role of aberrant neurodevelopment in schizophrenia were tested in the Methyl-Azoxy-Methanol (MAM) animal model. MAM treatment induces neurodevelopmental disruptions and behavioral deficits in off-springs of the treated animals reminiscent of those observed in schizophrenia and is thus considered a promising model for studying this pathology. On a gestational day-17, adult pregnant rats were treated with the antimitotic agent MAM. Experimental animals were divided into groups and subgroups according to substance treatment (MAM and vehicle agent [Sham]) and age of analysis (pre-adolescent and post-adolescent). Methylation and mRNA expression analysis of four candidate genes, which are often implicated in schizophrenia, with special emphasis on the Dopamine hypothesis i.e., Dopamine receptor D_2_ (*Drd2*), and the “co-factors” Disrupted in schizophrenia 1 (*DISC1*), Synaptophysin (*Syp)*, and Dystrobrevin-binding protein 1 (*Dtnbp1*), was performed in the Gyrus cingulum (CING) and prefrontal cortex (PFC). Data were analyzed to observe the effect of substance treatment between groups and the impact of adolescence within-group. We found reduced pre-adolescent expression levels of *Drd2* in both brain areas under the application of MAM. The “co-factor genes” did not show high deviations in mRNA expression levels but high alterations of methylation rates under the application of MAM (up to ~20%), which diminished in the further time course, reaching a comparable level like in Sham control animals after adolescence. The pre-adolescent reduction in DRD2 expression might be interpreted as downregulation of the receptor due to hyperdopaminergic signaling from the ventral tegmental area (VTA), eventually even to both investigated brain regions. The notable alterations of methylation rates in the three analyzed co-factor genes might be interpreted as attempt to compensate for the altered dopaminergic neurotransmission.

## Introduction

Schizophrenia is a severe neuropsychiatric disorder with a lifetime risk of 1% in the general population^1,2^ and implies a high social-economic burden for individual patients and society. Increasing evidence demonstrates that schizophrenia is a disorder of abnormal central nervous system (CNS) development^3–5^, with patients showing laminar disorganization, neuronal cluster in specific regions of the brain, and heterotopias^6–9^. Furthermore, there are also signs of neurodegenerative alterations in schizophrenia following its onset^10^. Therefore, schizophrenia has to be considered a multidimensional syndrome rather than a singular entity disorder^11^.

Functionally, at the endpoint of the pathophysiological cascade, when a schizophrenic psychosis is fully developed in individuals, a dopaminergic dysfunction is held responsible for the symptomatic presentation of the disease. At least, it is still thought to be one of the major contributors, as an increase in dopamine levels is one of the most consistent neurochemical abnormalities reported in schizophrenic patients^12^. Drugs that block dopamine D2 receptors are very effective in treating positive symptoms of schizophrenia^13^. Indeed, classical hypotheses imply that schizophrenic positive symptoms are mainly caused by dopaminergic over-activity of mesolimbic tracts^14^. Of the structures belonging to the limbic system, the cingulate gyrus (CING) has especially been implicated in the pathogenesis of schizophrenia^15^. Schizophrenic negative symptoms like attentional deficits, lack of insight into behavior, and others have been assigned to attenuation of dopaminergic function in the prefrontal cortex^16–18^, which is mainly based on the observation that neuroleptic drugs are not effective in treating negative symptoms. However, the discussion concerning the pathogenesis of negative symptoms is still controversial. There are some findings that could be interpreted in terms of reduced activation of Drd1 receptors in the prefrontal cortex (PFC), which could indicate a reduced input from the ventral tegmental area (VTA)^18^ while others found a reduced amount of Dopamine receptor D_2_ resp. D1 (*Drd2* resp. *Drd1*) receptors in this area^19^. However, is also known that the neuromodulator dopamine reduces PFC neuronal firing^20^, like recurrent excitations of glutamatergic circuits in the PFC, which are prerequisites for memory formation and abstract thinking^21^. Accordingly, in schizophrenic patients, a reduced prefrontal glucose metabolism has been confirmed in several functional neuroimaging studies using PET (positron emission tomography) or SPECT (single-photon emission computed tomography)^19^. Therefore, it cannot even be fully excluded that a dopaminergic over-activity of mesocortical tracts could account for negative symptoms.

The PFC is integrating sensory, motor, and affective data to shape planned behavior and shows a physiological loss of 30% of synaptic connections during adolescence in humans. In schizophrenia, the synaptic loss is even 60%, with adolescence in the same region^22^. In general, the pathogenesis of schizophrenia is thought to be a connectivity alteration^23^, with the main focus to date on frontal and cingulate cortices^15^. In line with this, both brain areas play an important role in information processing which is postulated to be disturbed in schizophrenia^15,24,25^.

Besides the dopamine receptor D2 (*Drd2*) itself, certain “co-factor” genes are prerequisites for functional neurotransmission, namely Disrupted in schizophrenia 1 (*DISC1*), Synaptophysin (*Syp*), and Dystrobrevin-binding protein 1, also known as dysbindin (*Dtnbp1*). These four genes are related to the functioning, formation, and preservation of dopaminergic synapses (as well as synapses in general) and have strongly been implicated in the pathogenesis of schizophrenia. *DISC1* has been shown to interact with a variety of molecules (described as “DISC 1 Interactome”^26^), thereby having a major impact on the regulation of neuronal developmental processes like the proliferation of neural precursor cells as well as the differentiation and neuronal axon/dendrite outgrowth in later developmental stages^27^. It is considered as a susceptible gene for schizophrenia, bipolar disorder, schizoaffective disorder, and it is also associated with disturbed cognitive functions^28^. Primarily, the gene has been discovered in a Scottish family, with 34 out of 77 family members having an abnormal translocation in chromosome 1. Sixteen of the affected family members had been diagnosed with psychiatric illness^27^ ranging from schizophrenia and major depression to bipolar disorder and adolescent conduct disorder^29^. In 2000, the gene was named “*DISC1*” describing the molecular nature of the mutation as the translocation directly disrupts the gene^30^. In mice, PFC, transient knockdown of the *DISC1* gene during pre- and perinatal stages has shown selective postnatal abnormalities in mesocortical dopaminergic neuron maturation accompanied by behavioral deficits which were likely to be triggered by disrupted post-adolescent cortical neuro-circuits^31^. *Syp* is a vesicle glycoprotein present in virtually all neurons and is known to regulate the trafficking of synaptobrevin and its retrieval after vesical fusion^32^ and is, therefore, directly related to synaptic function and turnover of neurotransmitters. It seems to be of special importance mainly during periods of increased and repetitive synaptic versicle turnover^33^ and exhibits strong homology between humans and rodents^34^. Although a study from 2000 could not detect any differences in the expression of *Syp* in postmortem PFC tissue between schizophrenic patients and controls^35^, most of the evidence reports significantly reduced levels of synaptophysin in the PFC of schizophrenic patients^36–40^. *Dtnbp1* has been shown to be localized, particularly in axon bundles and especially in certain axon terminals^41^, and to be involved in the transport of mitochondria to nerve terminals in order to preserve the presynaptic calcium homeostasis^42^. Thereby, it also plays a functional role in synaptic vesicle biogenesis and neurite outgrowth^43,44^ and has a regulative function on dopaminergic and glutamatergic neurotransmission^45^. *Dtnbp1* has significantly been associated with schizophrenia and bipolar disorder^46^.

In short, the three investigated “co-factor” genes for (dopaminergic) neurotransmission are important for the current functionality of synapses in the case of *Syp* (vesicle turnover) and *Dtnbp1* (maintenance of the calcium homeostasis) and for proper neuronal development resulting in dendrite outgrowth thereby creating prerequisites for synaptic connections and proper neurotransmission in case of *DISC1*.

The striking point in the pathogenesis of schizophrenia is, that the symptoms usually do not appear before but in/shortly after adolescence. One explanatory approach is the “dual hit hypothesis”, meaning that a certain inborn vulnerability, like subtle neuroanatomic lesions, has to be followed by an environmental trigger later on to initiate the occurrence of symptoms^47^. The effect of these environmental stimuli, i.e., aversive life events, cannabis use, or others, might be mediated by epigenetic changes in the promoter region of certain genes as epigenetic modifications are induced by the environment and can be stably preserved, thereby providing a link between inheritance and the environment^48^. Support for the hypothesis that epigenetics is involved in the pathogeneses of schizophrenia comes from the fact, that a sole genetic risk of schizophrenia is sceptical as the disease does not segregate in Mendelian’s manners^49^. DNA methylation occurs at cytosines positioned 5’ to guanosine (CpGs)^50^ and is one of the main regulatory epigenetic mechanisms^51^. This modification, in most cases, impairs transcription factor binding in the CpG-rich promoter region of a gene^52^, thereby leading to lower transcription. However, it still remains elusive why susceptibility to environmental cues is especially high during adolescence. One possible explanation could be that the extensive hormonal changes that take place during this time could cause greater changeability of methylation patterns than at other stages of life^53^. In general, adolescence is a unique developmental period in which social, emotional, and cognitive behaviors change dramatically^54–56^. Behavioral changes are often attributed to the neurodevelopment that occurs during adolescence, including myelination, synaptic pruning, changes in receptor levels, and projection elaborations, particularly in the PFC regions regulating cognition and inhibitory control^57–59^.

Access to postmortem brain tissues of individuals suffering from psychiatric disorders is a challenging task, and measurement of peripheral methylation does not always reflect the central activity of genes. In highly complex neuropsychiatric diseases like schizophrenia, animal models can contribute to a better understanding of the underlying pathophysiology. Currently, a variety of animal models are available to study the neurobiological mechanism of schizophrenia^60^.

In the current study, the MAM-based animal model was used, which is a highly reproducible and reliable model of schizophrenia^61,62^. The administration of MAM on embryonic day-17 (E17) interferes with the development of various brain regions, especially the PFC^63^, as neurogenesis peaks on E17^64^. While administration on E15 leads to dramatic gross brain abnormalities with major impairment of spatial learning, administration on E17 leads to aberrant cell migration with cognitive and working memory deficits^65^ as well as positive and negative symptoms like behavior^66^. The artificially induced neurodevelopmental abnormalities by MAM administration on E17 resemble those observed in schizophrenic patients’ brains^67,68^. MAM is a potent alkylating substance that methylates DNA at the N7 position of guanines^69^, has an anti-proliferative and antimitotic effect selectively on neuroblasts, and therefore interferes with the proper functioning of the central nervous system^70^. Administration of MAM during gestation has been shown to downregulate many genes that are critical for neuronal plasticity and neuron development in the off-springs^63,71,72^. Exposure to MAM during embryonic development disturbs specific brain region development in the off-springs^63,73–75^. Up to now, most epigenetic studies on DNA-Cytosin-methylation in the MAM model focused on the cannabinoid receptor 1 (CNR1)^76,77^ or used MeDip (Methylated DNA immunoprecipitation) for a broader analyses^78^, which is not always easy to be interpreted for statistical reasons. Other epigenetic studies investigated histone modifications^79–81^.

MAM treatment does not alter the litter size, gestational period, and body weights of adult rats. Behavioral abnormalities of MAM-treated animals include a deficit in prepulse inhibition (PPI) of startle, hypersensitivity to amphetamine, sensorimotor gating, social withdrawal, and an increased amphetamine hypersensitiveness during adulthood. All in all, behavioral changes mimic positive, negative, and cognitive symptoms, resembling findings in human schizophrenia patients^66,82,83^.

This study aimed to address two questions: (1) Are alterations in epigenetic regulation and expression of genes related to dopaminergic neurotransmission as well as to synapse function and preservation in PFC and CING involved in pathogenesis of schizophrenia? (2) Are there any time-course-related changes during adolescence in epigenetic regulation of schizophrenia candidate genes involved in dopaminergic neurotransmission and synapse preservation in the “default” circuits for schizophrenia, i.e., in the CING as part of the limbic system, and the PFC in the MAM rat model of schizophrenia?

## Results

In the MAM group, six rats were included in both, the pre-adolescent and post-adolescent groups. For the control arm of this study (Sham group), seven rats were included in both groups. Mean methylation rates and mRNA expression levels of the candidate genes *Drd2, DISC1, Syp,* and *Dtnbp1* were determined and correlated. Analysis was performed in the cingulum cortex (CING) and in pre-adolescent samples of the prefrontal cortex (PFC) (see also methods part for further explanation).

As data are relatively complex, we present results by relating MAM effects to the initial situation (Sham pre-adolescent group) first and subsequently mention the influence of adolescence in the respective text sections for every gene.

### Cingulate gyrus (CING)

**Fig. 1:**
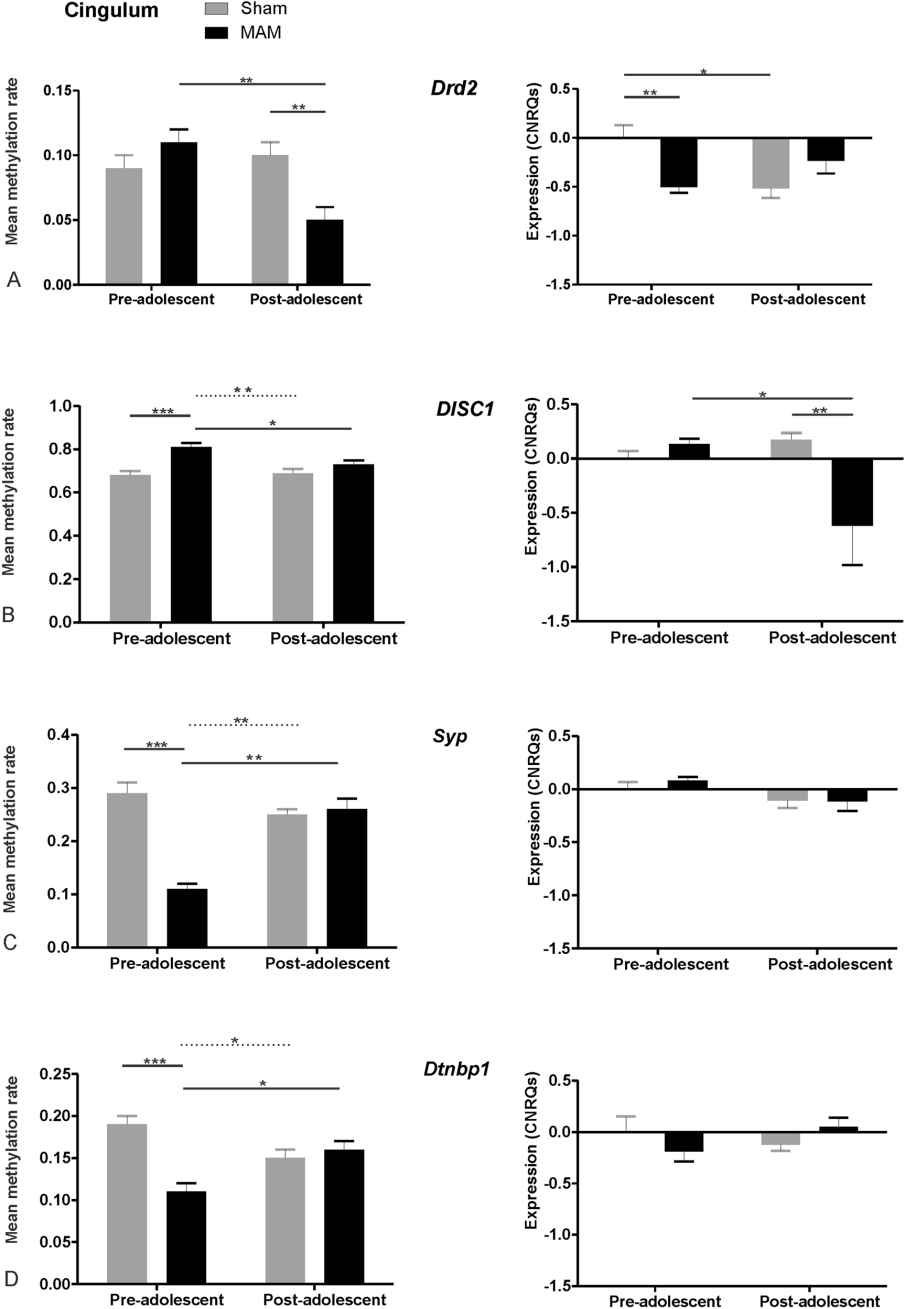
Mean methylation rates and expression levels (CNRQs, calibrated normalized relative quantities) of MAM and Sham pre-adolescent groups for the four investigated genes in the PFC. **A** Mean methylation rates and expression levels of Drd2, **B** mean methylation rates and expression levels of DISC 1, **C** mean methylation rates and expression levels of Syp and **D** mean methylation rates and expression levels of Dtnp1. Important significant differences between two groups are indicated by a line. Less important significant results (as the groups are not directly related) are indicated by dashed lines. The number of asterisks indicates the level of significance: *p < 0.05, **p < 0.01; ***p < 0.001. MAM animals were treated with Methylazoxymethanol-Acetate, Sham animals were only treated with vehicle. *Drd2* dopamine receptor 2, DISC1 disrupted in schizophrenia 1, Syp synaptophysin, *Dtnbp1* dysbindin. For the analysis 7 pre-adolescent Sham, 6 pre-adolescent MAM, 7 post-adolescent Sham and 6 post-adolescent MAM animals have been included.

Pre-adolescently, mean methylation rates for the *Drd2* promoter fragment did not show any significant effect of substance treatment. However, there was an effect of MAM treatment post-adolescently: the MAM post-adolescent group exhibited significantly lower mean methylation (Mean = 0.05, SEM 0.01, *N* = 6) than the Sham post-adolescent group (Mean = 0.10, SEM 0.01, *N* = 7), *p* < 0.01. Additionally, we found an effect of adolescence as the mean methylation was significantly lower in the MAM post-adolescent group as compared to the MAM pre-adolescent group (Mean = 0.11, SEM 0.01, *N* = 6), *p* < 0.01 (Fig. 1A, on the left).

In contrast, relative mRNA expression was already significantly altered pre-adolescently as in the MAM group relative expression (Mean = −0.50, SEM = 0.06, *N* = 6) was significantly lower as in the Sham group (Mean = 0.00, SEM = 0.13, *N* = 7), *p* < 0.01. Looking at the control group, we observed a lower mRNA expression in the Sham post-adolescent group (Mean = −0.51, SEM = 0.09, *N* = 7) compared to the Sham pre-adolescent group, *p* < 0.05. No other statistically significant differences were observed (Fig. 1A, on the right).

In the same brain region, the mean methylation of the *DISC1* promoter fragment was significantly higher in the MAM pre-adolescent group (Mean = 0.81, SEM = 0.02, *N* = 6) in comparison to the Sham pre-adolescent group (Mean = 0.68, SEM = 0.02, *N* = 7), *p* < 0.001. The mean methylation was also significantly higher in the MAM pre-adolescent group as compared to the MAM post-adolescent group (Mean = 0.73, SEM = 0.02, *N* = 6), *p* < 0.05. We did not observe any effect of substance treatment post-adolescently as methylation rates turned out to be comparable after adolescence between MAM post-adolescent group and the Sham post-adolescent group (Fig. 1B, on the left).

Relative mRNA expression of *DISC 1* did not show a significant effect of substance treatment between pre-adolescent groups. We could observe a significantly higher mRNA expression in the MAM pre-adolescent group (Mean = 0.13, SEM = 0.04, *N* = 6) in comparison to the MAM post-adolescent group (Mean = −0.61, SEM = 0.36, *N* = 6), *p* < 0.05. The MAM post-adolescent group has also shown significantly lower mRNA transcripts levels in comparison to the Sham post-adolescent group (Mean = 0.17, SEM = 0.06, *N* = 7), *p* < 0.01. No other statistically significant differences were found in expression analysis (Fig. 1B, on the right).

In the CING, the mean methylation rates of the *Syp* promoter fragment were significantly lower in the MAM pre-adolescent group (Mean = 0.11, SEM = 0.01, *N* = 6) as compared to the Sham pre-adolescent group (Mean = 0.29, SEM = 0.02, *N* = 7), *p* < 0.001. MAM pre-adolescent group has also shown significantly lower mean methylation in comparison to MAM post-adolescent group (Mean = 0.26, SEM = 0.02, *N* = 6), *p* < 0.01. After adolescence, we could not observe any significant effect of substance treatment (Fig. 1C, on the left).

In contrast to the methylation, we did not observe any statistically significant differences in *Syp*-mRNA expression (Fig. 1C, on the right).

For *Dtnbp1*, we could observe a significant effect of adolescence and substance treatment in both groups. Methylation rates were significantly lower in the MAM pre-adolescent group (Mean = 0.11, SEM 0.01, *N* = 6) in comparison to the Sham pre-adolescent group (Mean = 0.19, SEM 0.01, *N* = 7), *p* < 0.001. Methylation rates were significantly lower in the MAM pre-adolescent group also in comparison to the MAM post-adolescent group (Mean = 0.16, SEM 0.01, *N* = 6) (Fig. 1D, on the left), *p* < 0.05.

No statistically significant differences were observed regarding the expression of *Dtnbp1* (Fig. 1D, on the right).

For the sake of completeness: differences between MAM pre-adolescent values and Sham post-adolescent findings in *DISC1*, *Syp,* and *Dtnbp1* were also significant as indicated by dotted lines in Fig. 1B–D. However, as these two groups are not directly related to each other and the values of the post-adolescent Sham animals were comparable to those of the MAM animals, we have not presented these differences in the text so as not to detract from the more advanced and meaningful results.

#### Correlation between expression and methylation (cingulum cortex)

A mixed linear model was performed to study the correlation between methylation and expression. *Drd2* has shown a significant negative correlation for the MAM pre-adolescent group and a significant positive correlation for the Sham pre-adolescent group. In the case of *DISC1*, the only positive significant correlation was observed in the Sham pre-adolescent group. *Syp* has shown a significant negative correlation between methylation rates and mRNA expression in both pre- adolescent groups. In contrast, a significant positive correlation was found for both post-adolescent groups. For *Dtnbp1*, a significant negative correlation was observed for both pre-adolescent groups. The Sham post-adolescent group has shown a significant positive correlation with the CING. All in all, 5 of 7 pre-adolescent significant correlations were shown to be negative, whereas all significant post-adolescent correlations were shown to be positive in CING (Table 1).

**Table 1:** Fixed effects calculated with SPSS for expression and methylation correlation estimates in cingulum cortex.

Estimates of fixed effects								
Area	Substance	Puberty		Estimate	Std. error	Sig.	95% confidence interval	
							Lower bound	Upper bound
Cingulum cortex (*Drd2*)	Sham	Pre-adolescent	Methylation	**0.222**	0.061	**0.000**	0.101	0.343
		Post-adolescent	Methylation	0.029	0.106	0.783	−0.180	0.239
	MAM	Pre-adolescent	Methylation	***−0.684***	0.115	**0.000**	−0.912	−0.456
		Post-adolescent	Methylation	0.017	0.128	0.890	−0.235	0.271
Cingulum cortex (*Disc1*)	Sham	Pre-adolescent	Methylation	**0.098**	0.034	**0.005**	0.0295	0.166
		Post-adolescent	Methylation	−0.339	0.254	0.183	−0.841	0.161
	MAM	Pre-adolescent	Methylation	−0.030	0.060	0.614	−0.149	0.088
		Post-adolescent	Methylation	−0.027	0.045	0.554	−0.117	0.063
Cingulum cortex (*Syp*)	Sham	Pre-adolescent	Methylation	***−0.150***	0.021	**0.000**	−0.192	−0.108
		Post-adolescent	Methylation	**0.383**	0.084	**0.000**	0.217	0.550
	MAM	Pre-adolescent	Methylation	***−0.222***	0.076	**0.004**	−0.372	−0.073
		Post-adolescent	Methylation	**0.157**	0.046	**0.001**	0.066	0.247
Cingulum cortex (*Dtnbp1*)	Sham	Pre-adolescent	Methylation	***−0.226***	0.026	**0.000**	−0.277	−0.174
		Post-adolescent	Methylation	**0.256**	0.066	**0.000**	0.124	0.387
	MAM	Pre-adolescent	Methylation	***−0.359***	0.121	**0.003**	−0.598	−0.120
		Post-adolescent	Methylation	0.049	0.030	0.103	−0.010	0.108

Dependent Variable: Normalized_relative_quantities. Significant findings are presented in bold print. Of those, negative correlation estimates are in italic.

### Prefrontal cortex (PFC)

In the case of the PFC, only pre-adolescent group samples were available due to technical reasons (as explained in the methods section). Therefore, pre-adolescent data are only shortly presented as an add-on, and the corresponding figure and table are given as a supplement.

We did not observe significant differences in the mean methylation (Supplementary Fig. 1, on the left) but a significantly lower mRNA expression level of *Drd2* in MAM animals (Mean = −0.47, SEM 0.13, *N* = 6) compared to Sham animals (Mean = 0.0, SEM 0.11, *N* = 7) (Supplementary Fig. 1A, on the right), *p* < 0.05.

Mean methylation of the *DISC1* promoter fragment was also higher in the MAM group (Mean = 0.82, SEM 0.01, *N* = 6) in comparison to the Sham group (Mean = 0.78, SEM 0.01, *N* = 7) (Supplementary Fig. 1B, on the left), *p* < 0.05.

No significant differences were observed in *DISC1*-mRNA levels (Supplementary Fig. 1B, on the right).

Mean methylation in the *Syp* promoter fragment was higher in the MAM group (Mean = 0.28, SEM 0.02, *N* = 6) in comparison to the Sham group (Mean = 0.16, SEM 0.01, *N* = 7) (Supplementary Fig. 1C, on the left), *p* < 0.001.

For *Syp*-mRNA transcripts, no significant differences were observed among substances (Supplementary Fig. 1C, on the right).

Methylation rates were significantly lowered in MAM-treated animals (Mean = 0.10, SEM 0.01, *N* = 6) in comparison to the Sham group (Mean = 0.15, SEM 0.01, *N* = 7) for the *Dtnbp1* promoter fragment, *p* < 0.01 (Supplementary Fig. 1D, on the left).

In contrast, we did not observe significant differences among *Dtnbp1*-mRNA levels (Supplementary Fig. 1D, on the right).

#### Correlation between expression and methylation (PFC)

A mixed linear model was performed to study the correlation between two variables (Supplementary Table 1).

In the PFC, significant positive correlations for *Drd2*, *Syp,* and *Dtnbp1* were found in the Sham pre-adolescent group between methylation rates and mRNA expression. Significant negative correlations were found for the MAM pre-adolescent group in the *DRD2 and Dtnbp1* genes.

All in all, in the PFC, the direction of significant correlations was positive in the Sham group, whereas it was negative in the MAM group.

## Discussion

In the present study, we aimed to investigate whether two main findings of schizophrenia research, namely the alteration in dopaminergic neurotransmission as well as the high vulnerability during adolescence as reflected by the time point of symptom-onset as well as by significantly higher post-adolescent loss of synaptic connections than in normal conditions on a molecular level^22^, might be based on adolescence-related alterations in epigenetics, i.e., in the changeability of methylation pattern.

Therefore, mean methylation rates and mRNA expression of genes involved in dopaminergic neurotransmission as well as in synapse formation, function, and preservation, namely *Drd2* and the “synapse-co-factors” *DISC1, Syp*, and *Dtnbp1*, were investigated in the cingulate and prefrontal cortex being in scope of current schizophrenia research^15^ and finally correlated with each other.

### Cingulate gyrus (CING)

In the case of CING, the fact that the two Sham groups (pre- and post-adolescent Sham group) show similar methylation levels facilitates orientation in this relatively complex set of data.

For *Drd2* (Fig. 1A), we could observe an effect of adolescence in the MAM group and of substance treatment post-adolescently. Although pre-adolescently, methylation rates were increased by MAM only in trend, expression of *Drd2* has been significantly reduced in the MAM pre-adolescent group. Indeed, the significant negative correlation for the MAM pre-adolescent group (Table 1) further indicates that changes in mRNA expression levels might have been mediated by alterations of CpG methylation in the *Drd2* promoter. After adolescence, there was a marked decrease in the rate of methylation related to MAM treatment (from 10 to 5%), and expression recovered, respectively, even though only by trend. As expression levels also decreased in the control group but only after adolescence, our findings suggest that the reduction in *DRD2* expression levels might be something normal but happened too early in MAM-treated animals. Therefore, the reduction of methylation over time (after adolescence) to a level even lower than that observed in the post-adolescent control group could be considered compensatory, resulting in a less reduced expression level. However, since brain development is strictly organized in a temporal-spatial manner, this compensation is probably too late to fully compensate for deficits.

It is very likely that the early reduction in DRD2 expression can be interpreted in terms of a downregulation of the receptor^84^ in response to excessive dopaminergic transmission/input into the CING from the VTA under MAM application. In the literature, there are already hints of a hyperdopaminergic system in MAM animals^85,86^. Transferred to human beings, this over-activation would correspond to an increased mesolimbic dopaminergic transmission underlying schizophrenic positive symptoms. The CING, in particular as an important part of the limbic system, is known to express significant amounts of D1 receptors^87,88^ besides a moderate expression of DRD2 receptors. Strongly simplified, activation of D1 receptors is known to induce excitatory potentials, whereas activation of Drd2 has inhibitory properties^89^. Due to the predominance of D1 receptors in this area, the excessive dopaminergic stimulation would lead to excitation in total, resulting in positive symptoms. Furthermore, in an examination of schizophrenic patients by positron emission tomography (PET), a lower binding potential of DRD2 receptors has been detected in the anterior CING^90^, being in line with our findings. Taking the downregulation of DRD2 as an indicator for increased mesolimbic dopaminergic neurotransmission, it can be assumed that the rat model mimics the situation in schizophrenia quite well.

As in *Drd2*, we could observe the effect of substance treatment in pre-adolescent groups and the effect of adolescence in MAM treatment in *DISC1* (Fig. 1B). Methylation was significantly higher in the MAM pre-adolescent group in comparison to the Sham pre-adolescent group and MAM post-adolescent group. However, changes in mRNA transcript levels were somewhat counterintuitive concerning the observed methylation pattern. Instead of lower expression in the MAM pre-adolescent group, the lowest expression was detected in the MAM post-adolescent group compared to both the pre-adolescent group of MAM-treated animals and the post-adolescent group of Sham. Also, there were no significant correlations besides the positive one in our pre-adolescent control condition (similar to the findings in Drd2). Reduced *DISC1* expression in the CING after adolescence might therefore be independent of the observed changes in the mean promoter methylation, although the observed significant differences between conditions were up to 13%. On the other hand, it might be possible that the effect of the pre-adolescent increase of methylation in MAM animals only becomes visible later or that the expression would have been even lower without the alterations in methylation rates. However, one could speculate that the increase of the *DISC1* methylation rate itself could be reactive toward the alterations of the dopaminergic system. As *DISC1* is known for its strong implication in neurite outgrowth, neuron positioning, dendritic development, and synapse formation^91^, increasing its methylation rate might be interpreted somewhat daringly as an attempt to decelerate development as errors in synaptic connections during development cannot be corrected at later developmental stages. In any case, the lower expression after adolescence in comparison to the control group indicates the incorporation of neurodevelopmental abnormalities in our animal model, eventually mediated by additional factors rather than the change of methylation rates only.

Concerning *Syp* (Fig. 1C), there was an effect of MAM treatment on methylation in the pre-adolescent group, leading to significantly lower methylation in MAM-treated animals. However, corresponding elevations in *Syp* mRNA levels did not reach significance. Nevertheless, we found significant negative correlations between the mean methylation rate and expression in both pre-adolescent groups and significant positive correlations in both post-adolescent groups. Significant correlations, on the one hand, but lack of significant group differences in the expression on the other, might be explained by the fact that alterations in methylations are mostly more stable than respective RNA levels and can therefore be detected more reliably. Furthermore, it would be conceivable that we have caught a point in time where we could only observe the first step, namely the enormous reduction of the methylation rate (−18%), creating prerequisites for a compensatory reaction towards the changes in the dopaminergic neurotransmission by making the DNA more accessible for potential transcription signals. *Syp* is a molecule, which is related to synaptic activity as it is a vesicle protein. Therefore, it is conceivable that due to the increased dopaminergic stimulation of neurons in the CING their firing rate is rising, thereby increasing the need for vesicle proteins, which is reflected in reduced methylation rates. As *Syp* is concerned with the current functionality of synapses, it needs to be regulated faster than Drd2 where we observed the potential compensatory reaction (regarding methylation) later (after adolescence).

In *Dtnbp1* (Fig. 1D), we also observed a significant effect of substance treatment and adolescence. There was a significant pre-adolescent decrease (−8%) of methylation rates in MAM animals compared to Sham pre-adolescent animals, as we saw in *Syp*. Although there has been no corresponding elevation in transcript levels, we found a significant negative correlation of mean methylation rates with the expression level in the MAM pre-adolescent group. As for the other “co-factors” it might be argued that the reduction of methylation rate might be reactive towards the alterations in dopaminergic neurotransmission. *Dtnbp1* has been shown to have a regulative function on dopaminergic and glutamatergic neurotransmission^45^. As *Dtnbp1* is known to transport mitochondria to nerve terminals in order to preserve the presynaptic calcium homeostasis^42^, it is also conceivable that the demand for this molecule is higher in the presumed state of higher excitation.

Overall, regarding alterations in pre-adolescent methylation levels, the application of MAM had a significant impact on all Drd2-“co-factor-genes” (not DRD2 itself) in the CING, as indicated by significant and really high pre-adolescent differences in methylation rates between Sham and MAM animals, which have most probably to be considered as preparation to adapt to the altered dopaminergic neurotransmission as discussed above. In addition, adolescence seems to play an important role, although almost exclusively in MAM animals, as there were significant differences between pre- and post-adolescent methylation in MAM animals in all four genes. Roughly, one could say, that the previous pre-adolescent consequences of prenatal MAM application are somewhat counteracted after adolescence, independent of their initial direction (Fig. 1) as indicated by comparable methylation levels in post-adolescent MAM- and Sham animals. Even though the directions of the alterations differed between genes, this clearly indicates the impact of MAM as there were almost no alterations between the different time points in the Sham group. In short summary, MAM had the greatest impact on the CING in pre- adolescence, which was attenuated as development progressed.

With regard to expression, the main findings were a significant reduction of pre-adolescent Drd2 expression and post-adolescent *DISC1* expression under the application of MAM. The reduction in Drd2 expression might be interpreted as a downregulation due to excessive dopaminergic stimulation from the VTA. Therefore, the alterations of pre-adolescent *DISC1*, *Syp,* and *Dtnp1* methylation rates under MAM treatment appear to be kind of adaptive. Although there were only small alterations in the expression of the “co-factor” genes, we could observe the same general principle as with the alteration of methylation rates, i.e., the changes due to MAM were counteracted by time, and in part, they were even overcompensated for.

The temporary appearance of altered methylation rates shortly before adolescence might reflect/mirror the higher vulnerability in schizophrenia as they possibly provide the basis for severe consequences of a “second” hit in adolescence. Without a second hit, like in our case, expression levels might be less affected and the whole system can even recover to a certain extent.

However, even though methylation rates/expression levels normalized after adolescence, it is conceivable that temporary pre-adolescent alterations already led to an altered architecture of the CING and additionally might have had an impact on the development of other brain areas due to the complex connectivity of all brain regions, accounting for the phenotypic changes seen in MAM animals^61,63,65,66^.

Furthermore, it might be possible that the current consequences of altered methylation rates on expression would have been clearer if protein levels were also considered instead of just mRNA levels.

An additional interesting observation was that correlation analysis between methylation and mRNA levels revealed different patterns for the two time points (Table 1). When looking at the significant correlations between methylation level and expression in the CING for every single condition, the pre-adolescent conditions showed predominantly negative correlations (5/7), while in post-adolescent conditions, all (3/3) correlations were positive. During development, positive correlations do not seem to be unusual. For instance, Merid et al.^92^ were able to show in human blood from children (age 0–18 years) that the correlations were positive for 54% of those genes that showed a significant correlation of methylation and expression. However, concerning our main findings, correlations were classically negative, meaning that a lower methylation rate leads to a higher expression of respective genes. In case of no correlation, other epigenetic factors like histone modifications could be involved.

### Prefrontal cortex (PFC)

Only pre-adolescent samples were available for the PFC analysis, revealing no significant differences between groups for *Drd2* methylation analysis (Supplementary Fig. 1A). However, the MAM group exhibited a significantly lower expression of *Drd2* than the Sham group, and a significant negative correlation for the MAM group was observed.

The lower expression of Drd2 is in line with some earlier findings from other groups^27^. Up to now, it is not clear why the number of receptors is reduced in the PFC in schizophrenia. A bit daringly, one could even think of a compensatory downregulation of the receptor due to overstimulation of the PFC by dopaminergic afferents from the VTA, although one spread explanatory approach for negative symptoms assumes a lack of activation from the VTA. However, so far, this has not yet been conclusively proven, and findings in this regard are contradictory^93^. Considering the regional molecular distinctions, the consequence of dopaminergic overstimulation in the PFC is different from that in the CING: as described above, higher dopaminergic input into this region leads to a reduction of neuronal firing in the PFC like recurrent excitations of glutamatergic circuits, which is prerequisite for adequate cognitive functioning^20^.

This might be due to the fact that the ratio between *Drd2* and *DRd1* receptors is different in both brain areas in adults with more comparable levels of both receptor subtypes in the PFC^94^ in contrast to the CING with a predomination of *Drd1* receptors^87,88^. Therefore, *Drd2* has a greater impact in the PFC than in CING, and activation of *Drd2* is considered to mainly exert inhibitory effects on the target neuron. Additionally and probably even more crucially, although excitatory effects have been assigned to *DRD1* in general^89^, it has been shown that specifically in the PFC, their activation produces an “inverted-U-shaped dose-response curve”. This means that moderate levels of activation improve PFC function, while high levels lead to a significant impairment^95^. The reduction in PFC neuronal firing by high activation of DRD1 is most likely mediated by the opening of hyperpolarization-activated cyclic nucleotide-gated cation (HCN) channels at excitatory synapses leading to reduced cognitive performance^96^. HCNs are mainly regulated by intracellular cyclic nucleotides like cAMP and cGMP, lowering their threshold potential. It is worth mentioning that HCNs are permeable not only for potassium but also for calcium ions, so the opening of these channels results in significant intracellular calcium peaks^97^.

For *DISC1* (Supplementary Fig. 1B), we observed significantly higher methylation in the MAM group (+4%). Although expression levels were comparatively lower in comparison to the Sham group, differences did not reach significance. It might be speculated that, as observed in the CING the increase of methylation rates is reactive towards the alterations in dopaminergic neurotransmission in order to prepare for a deceleration of the developmental process.

In *Syp* (Supplementary Fig. 1C), the significantly higher methylation in the MAM group has also not been accompanied by significant changes in mRNA levels. There was also no significant correlation between mRNA expression and DNA methylation for any group. In contrast to our findings in the CING we found an upregulation of the methylation rate (+15%), which has to be interpreted as an attempt to silence *Syp* expression. As mentioned above, dopaminergic projections from the VTA to the PFC are known to decrease neuronal firing in the PFC, which has been attributed to negative symptoms. Therefore, a decreased need for *Syp* is reasonable, assuming decreased neurotransmission in the PFC in the MAM-treated animals.

In line with this, there are many reports showing reduced levels of *Syp* in PFC areas in patients with schizophrenia^36,98–100^. One study reported unchanged *Syp* messenger RNA in the PFC of schizophrenic patients^101^, which might be due to the different time points of analysis.

Methylation rates of *Dtnbp1* (Supplementary Fig. 1D) in the MAM group were significantly lower in comparison to the Sham group (−5%), which is quite a solid difference. There were no significant alterations in mRNA levels, but we observed a significant negative correlation for the MAM pre-adolescent group in further correlation analysis between expression and DNA methylation in the PFC.

Again, the change of methylation rates might be interpreted as preparation for adaption to the altered dopaminergic input to the PFC in terms of increased provision of *Dtnbp1*. As mentioned before, this molecule regulates synaptic function by preserving the calcium homeostasis^42^ at the end terminal of axons, ensuring the proper functioning of synapses and neurotransmission. Although the neuronal firing rate is generally reduced in the PFC, there may be nonetheless an increased need for regulating calcium homeostasis if, for example, inhibitory effects are caused by the opening of HCNs, as described above, which can also lead to an excessive influx of calcium ions. As stated before, is of utmost importance to preserve a proper level of intracellular calcium concentrations, as calcium signals need to be very precise in a temporospatial manner^102^. Therefore, it is explainable why despite distinct effects on activity in the two investigated brain areas, the altered dopaminergic input can lead to an increased need for dysbindin in both conditions.

However, the higher readability of the promoter sequence was apparently not exploited in the MAM animals (at least as indicated by unaffected mRNA levels). Nevertheless, in case we would have applied a “second hit” in our model, these alterations in methylation rates might have been used and consolidated. Furthermore, it might also be that we caught a time point where we could only see the preparations for the compensatory increase.

We also had a look at the distribution of significant correlations in the PFC as we did in CING (Supplementary Table 1). It became apparent that in PFC, pre-adolescent Sham groups (of different genes) showed positive correlations (3/3), whereas there were only negative correlations in MAM-treated animals (2/2).

Considering pre-adolescent findings of the CING we had a mixture of positive (2/4) and negative correlations (2/4) in the Sham groups, and in accordance with the PFC, only negative correlations in MAM-treated animals (3/3). However, significant findings in methylation or expression were always associated with significant negative (or else insignificant) correlations.

To our knowledge, most publications concerned with epigenetics in the MAM rat model investigated histone modifications^79–81^ so far. Only a few investigated DNA methylation in this context: two of them were focusing on CNR1-promotor-methylation^76,77^, whereas another study used MeDip for broader analyses^78^, which is not always easy to be interpreted for statistical reasons. Therefore, the present study is the first study investigating the role of DNA methylation in the MAM rat model over the time course of adolescence in synapse-related genes.

## Summary and conclusion

Our findings clearly indicate that the application of MAM led to reduced expression levels of *Drd2* in pre-adolescence in both brain areas, CING and PFC. Interestingly, time could partly compensate for this effect as shown by restored expression levels after adolescence. The reduced pre-adolescent expression of *Drd2* in both investigated brain areas might be interpreted as downregulation, eventually indicating dopaminergic overstimulation from the VTA, which is in line with the dopamine hypothesis of schizophrenia, at least in the case of CING.

The “co-factors” for functional neurotransmission showed notable alterations of their promoter methylation rates with differences as big as up to almost 20% at the same time point (pre-adolescent), which is probably to be interpreted as an attempt to adapt towards the altered dopaminergic input from VTA. The direction of the alteration was concordant for both brain areas with regard to *DISC1* and *Dtnbp1*. *DISC1* methylation rates were increased, eventually indicating a pursuit to decelerate further neurite outgrowth under pathological conditions. *Dtnbp1* methylation rates were reduced probably in order to sustain calcium homeostasis at the axon endpoints, which is challenged in both brain areas despite opposing effects of the dopaminergic input on neuronal firing due to locally different predominant composition and functionality of receptors. *Syp* methylation rates were reduced only in CING, indicating higher synaptic activity induced by dopaminergic overstimulation. In contrast, it had increased in the PFC. As known, there is a so-called “hypofrontality” in schizophrenia, and dopaminergic stimulation has an inhibitory effect on neuronal firing in the PFC^20^, leading to a reduced need for vesicle proteins like *Syp*.

It could be argued that the alterations in methylation rates were directly induced by MAM. However, MAM is known to methylate Guanin bases and not cytosins, which have been analyzed here. Furthermore, in case it would be a direct effect of MAM the question would arise why changes had different directions. Furthermore, the time course would not fit this hypothesis.

All in all, our findings strongly support the classical dopamine hypotheses as part of a complex interplay between different brain regions and neurotransmitters in the pathogeneses of schizophrenia in the case of CING. In the case of PFC our results might, aberrantly from the mainstream assumption so far, point to increased dopaminergic input from the VTA. However, this is only a small study, and this interpretation needs to be further investigated. The alterations occurred as early as already pre-adolescently in the MAM model we used. In the further course, findings mitigated after adolescence. The pre-adolescent alterations in methylation might be interpreted as a preparation to adapt to altered dopaminergic input and might provide the basis for the typically increased vulnerability during adolescence to a “second hit” as the accessibility of the DNA for transcription factors is altered. However, even without a “second hit” MAM animals show already phenotypic alterations, which might be due to the fact that already temporal alterations during development might lead to severe permanent anatomical and functional pathologies^37,61,63,66^ due to the interconnectivity of brain regions and the strict temporospatial organization of brain development.

It might also be possible that we simply missed minute changes in mRNA levels^103^ resulting from the huge presumably adaptive alterations of methylation rates which are undetectable with current methodologies, but which could significantly impact protein expression. Future research is also required to clarify if mRNA and protein levels have a direct correlation in the investigated conditions and time points.

Additionally, we could support findings from Merid et al.^92^ that the correlation between expression and methylation levels of a gene are often positive in younger age. However, the correlation was classically negative in conditions where we detected significant alterations in either methylation or expression.

### Limitations

For technical reasons, it was not possible to extract proteins from the samples, which would have been helpful for a better categorization of our findings. It would have been also desirable to have data from the post-adolescent PFC. Also, it would have been interesting to study DRD1. The existing literature supports the usefulness of the MAM model in schizophrenia research. There are consistent findings regarding disrupted hippocampal, midbrain, and striatal interconnectivity, and altered glutamatergic and dopaminergic function. However, neuroanatomical variations between species (human and rodent) should be always considered, and for ethical reasons, alternatives to animal testing are always desirable. Another limitation is that we had a very small part of Cg1 within our PFC preparation for stereotaxic reasons. Although it was negligibly small, a slight influence on the PFC data cannot 100 % be excluded.

## Materials and methods

### Animals

In the working group of Marie-Odile Krebs, INSERM (Institut National de la Santé et de la Recherche Médicale), Paris, France, pregnant Sprague-Dawley dams (Charles River, France) were obtained at day 10 of pregnancy. On E17 they had been treated with either MAM (25 mg/ml/kg, MAM-treated group) or vehicle agent (NaCl 0.9% 1 ml/kg, Sham group) to model schizophrenia^43,60,104^. Male off-springs were weaned on day 21 and kept in groups of 2–3 rats with 12 h light/dark cycle while water and food were provided ad libitum. These rats were sacrificed either on day 23 (pre-adolescent group) or day 63 (post-adolescent group) via CO_2_ inhalation^70^.

We use the term “post-adolescent” instead of “adult” to indicate the very early stage of adulthood/late adolescence. The length of adolescence in the rat has been suggested to subsume from a conservative 2 weeks (e.g., postnatal days [P] 28–42^105,106^) to a broader time span of 6 weeks (e.g., P25–65)^106^. The latter age span is thought to encompass the range from early adolescence through late adolescence/emerging adulthood and has been parsed into subgroups in various ways^106,107^. For example, the P25–42 interval in rats may be roughly analogous to the 10–18 year, the early-mid adolescent period in humans, with the ages from P43 to P55 or P65 approximating the 18–25-year-old period of late adolescence/emerging adulthood in humans^106^.

Brains of the animals were isolated and divided into different anatomical regions utilizing an Adult Rat Brain Slicer Matrix, immediately frozen after dissection, and were sent to LMN, Hannover Medical School, Germany.

The prefrontal cortex corresponds mainly to the medial prefrontal cortex with a tiny part of cingulate area 1 (compare rat brain stereotaxic coordinates: interaural 12.72 mm, Bregma 3.72 mm). The gyrus cingulum corresponds to the cingulate area 1 and cingulate area 2 on a slice posterior to the one where PFC was sampled (compare rat brain stereotaxic coordinates: interaural 11.28 mm, Bregma 2.28 mm).

Unfortunately, the post-adolescent samples of the PFC had been stored in a second box for reasons of space. When samples were sent to Hannover the second box was sent separately later. Due to a delay during shipping, samples in the second box thawed and could, therefore, not be included in the further analysis. Therefore, we initially thought to skip this brain region completely. However, as there is still some important information in the pre-adolescent samples, we finally decided to include the data in our manuscript, at least as a supplement.

### DNA and RNA extraction

Tissue samples were thawed, and a maximum of 10 mg of tissue was used for the following procedure. AllPrep 96 DNA/RNA kit (QIAGEN) was used for DNA/RNA extraction according to the manufacturer’s protocol. Therefore, DNA and RNA are coherent samples, i.e., originating from the same animals. DNA was stored at −20 °C, and RNA was stored at −80 °C after isolation until bisulfite conversion or cDNA transcription for further analysis was performed. Quantification of DNA and RNA was performed spectrophotometrically with the Nanodrop-1000 (Thermo-Scientific®).

### Methylation analysis

Bisulfite treatment of DNA enables methylation analysis of DNA fragments. During bisulfite treatment methylation prevents the conversion of cytosine to uracil. Therefore, methylated cytosines can be identified as cytosines, whereas unmethylated ones occur as thymines compared to the original genomic sequence. In the present study, the EpiTect 96 Bisulfite Kit (QIAGEN) was used for bisulfite conversion. The standard bisulfite reaction mixture was prepared with 500 ng DNA at room temperature. The thermal cycler was set according to the manufacturer’s protocol.

Bisulfite treatment results in a less specific DNA strand for the primers. To compensate for lower specificity, (semi-)nested-PCR was performed in two successive runs with two different sets of primer pairs (one outer primer pair and one inner primer pair). Nested-PCR was followed by an automated purification of the PCR product on a Biomek^(R)^ NX^P^ Roboter (Beckman Coulter ®) by the use of AMPure XP paramagnetic beads (Agencourt®).

Sequencing was performed according to a Sanger 3500xL Genetic Analyzer (Applied Biosystems®). For this purpose, the sequencing-PCR was prepared to utilize the Big Dye Sequencing Kit (Applied Biosystems®), and the product was purified by using CleanSeq paramagnetic beads (Agencourt®) on a Biomek^(R)^ NX^P^ Roboter (Beckman Coulter). Primers are to be found in Table 2.

**Table 2:** Gene names, symbols and primer sequences of bisulfite primers.

Gene	Gene symbol	Primer sequences	Number of CpGs analyzed	Genomic position of mapped target (mRatBN7.2 ref., annotation release 108)
Synaptophysin	*Syp*	Fw_o: 5’TTTAGTTGGGTGATTTTGTG3’ Fw_i: 5’AGTTTTTAGGATGGAATGGT3’ Rev_o: 5’CTCTATTTCCTAACTCATCTA3’ **Rev_i:5’ACATCTAAAAACCCCCTCT3’**	36	Chr. X: 14864747 to 14864282 (area includes exon 1)
Disrupted in schizophrenia 1	*Disc1*	Fw_o: 5’ATGGGGTATTTTTTTGTTTTT3’ Fw_i: 5’AAAAGGGGTGGAAAGGT3’ Rev_o: 5’AAATCAAAACCATAACTAAAC3’ Rev_i:5’CAAAAAAAAATAACCCAAATC3’ **Rev_sequencing: 5’TACCAAAATAACTAAAATAAAT3’**	23	Chr. 19: 53046841 to 53047303 (area positioned in 5‘ direction from exon 1)
Dopamine receptor D2	*Drd2*	Fw_o: 5’TAGAGTAGGTTTGGATGGGTAGG3’ Fw_i: 5’GGTTTTTTTTTTAGGTTTTAT3’ Rev_o: 5’AAAAAAACTAAAAATAACATCCTTCA3’ **Rev_inner:5’ACTAAACAAACTTCTATTCCTAAC3’**	62	Chr. 8: 49708758 To 49709332 (area includes exon 1)
Dystrobrevin binding protein (Dysbindin)	*Dtnbp1*	Fw_o: 5’GGGTTTATTAGTTGAGGGTT3’ Fw_i: 5’AAGGTTGGGTTTGTAAGGAT3’ Rev_o: 5’CTAACTCCGATTCCTACAC3’ Rev_i:5’CACCTTCCCAAAAAACTAAC3’ **Rev_sequencing: 5’CCAATAACAAAAAAAAAAACTA3’**	71	Chr. 19: 51548644 to 51548148 (area includes exon 1)

Amplification was done by nested PCRs in order to compensate for the lower specificity of the DNA sequence after bisulfite conversion. In the first round of the nested-PCR, the “outer” primers were used (abbreviation “o” in the primer names), in the second round the inner primers (“i”). The respective sequencing primer for each gene is given in bold print.

Primers were designed with the help of the software “Geneious”, which can also be used to identify “CpG-islands” within gene promoters. Sequence segments were defined as CpG-islands when containing at least 60% CpGs. The genomic sequence was converted into a bisulfite sequence by the use of Methyl-Primer-Express in order to design bisulfite primers. Primers were located around the CpG-islands and had to fulfill different criteria (no CpGs in the primer sequence, comparable melting temperatures, length between 18 and 32 base pairs etc.).

### mRNA expression analysis

RNA was reversely transcribed by using the iScript Kit (Bio-Rad®). The reaction mixture and thermal cycler program were following the manufacturer’s protocol.

For RT-qPCR, primers were established, taking the following considerations: at least one primer (of each primer pair) was positioned on the exon-exon boundary to bypass genomic DNA. Primers were designed to have a product size between 150–300 base pairs. Only those primer pairs were accepted, which were free from self-dimers, hairpins, and cross dimers (as checked by NetPrimer software). The target-specificity of the primers was checked using the BLAST database. Only target-specific primers were accepted for further experiments. Only those primer pairs were utilized, which had assay efficiencies between 90 and 105%.

GoTaq® qPCR Master Mix kits (Promega®) were used to perform real-time PCR, and the standard cycler program for the kit was followed.

Primer sequences, type of gene (target genes = TG and reference genes = RG), position according to exons, and chromosome where the target is located is given in Table 3. The optimal temperature for amplification, and efficiencies for primer pairs is illustrated in Table 4.

**Table 3:** Gene names, symbols and primer sequences, type of gene (TG = target gene, RG = reference gene), and position (Exon-exon borders) of the real-time primers as well as the respective chromosome on which the gene is located.

Gene	Gene symbol	Primer sequences	Type	Location E = Exon	Chromosome
Synaptophysin	*Syp*	Fw: 5’TAGCCAGAAAGTCCATCATA’3 Rev: 5’CAAAGGGGGCACTACCAA3’	TG	E3-E4, E4	X
Disrupted in schizophrenia 1	*Disc1*	Fw: 5’CGCCCACAAAACACGC3’ Rev: 5’TA GGG ACA GCC AGG G3’	TG	E8-E9, E10	19
Dopamine receptor D2	*Drd2*	Fw: 5’CTGGAGGTGGTGGGT3’ Rev: 5’CA GTG GGC AGG AGA TG3’	TG	E2-E3, E5	8
Dystrobrevin-binding protein (dysbindin)	*Dtnbp1*	Fw: 5’ATGCTGTCTGCCCACTG3‘ Rev: 5’GCTCCTTCCTCTTACTTTTCT3‘	TG	E5, E6-7	17
Glyceraldehyde-3-phosphate dehydrogenase	*Gapdh*	Fw: 5´TGC CAG CCT CGT CTC ATA G3’ Rev: 5’CTT CCC ATT CTC AGC CTT G3’	RG	E1-E2, E3	4
Succinate dehydrogenase complex, subunit A	*Sdha*	Fw: 5’GCAGCACAGGGAGGTATCAATG3’ Rev: 5’GCTCAACCACGGAGGCAGGA3’	RG	E3-E4 E4-E5	1
ß-actin	*Actb*	Fw: 5’CGCCACCAGTTCGCCAT3’ Rev: 5’CTGACCCATACCCACCAT3’	RG	E1-E2, E3	12

**Table 4:** Primer PCR specifications.

Gene	Optimal PCR temperature (°C), (X°C)	Primer efficiency
Synaptophysin	57.7 °C	1.986
Disrupted in schizophrenia 1	53.5 °C	1.970
Dopamine receptor D2	56.9 °C	1.933
Dystrobrevin binding protein or dysbindin	57 °C	1.917
Glyceraldehyde-3-phosphate dehydrogenase	60.7 °C	1.923
Succinate dehydrogenase complex, subunit A	54.8 °C	1.956
ß-actin	62.5 °C	1.908

### Statistical analysis

Further analysis of methylation data was performed using the ESME (Epigenetic Sequencing Methylation Analysis Software) software^108^ and SPSS (Statistical Package for the Social Sciences, Version 24, Chicago, Illinois). Methylation rates of all the CpGs analyzed in the fragment were chosen as descriptive variables, and different treatments were chosen as factors for the model. CpGs with more than 5% of missing values, higher standard deviation (5%), and higher variance (0.1%) were excluded from the analysis. The first base of the first exon corresponds to position zero. For further statistical analysis by a mixed linear model, the ESME results were exported to IBM SPSS Statistics. Dataset representation was performed in GraphPad PRISM 9.

For mRNA expression analysis, post-qPCR quantification summaries from the cycler were exported to qBase software. This software uses an algorithm for the calculation of calibrated normalized relative quantities (CNRQs), thereby already considering the data from the reference genes. The algorithm is also reflecting inter-run variation and other important aspects that have to be taken into account for a reliable analysis of qPCR results. The CNRQs were exported to SPSS, and mean values were calculated for CNRQs and Standard errors of the mean. The dataset was exported to GraphPad PRISM for final representation and statistics.

Relative quantitative values were normalized to the three reference genes Gylceraldehyde-3-phosphate dehydrogenase (GAPDH), succinate dehydrogenase (SDHA), and Beta-Actin (ACTB). Samples were always run in triplicates. Higher expression results in a positive ∆Cq, and lower expression results in a negative ∆Cq.

Statistical analysis was performed in Prism using two-way ANOVA for the CING. For the PFC region *t*-test had to be applied as there were only pre-adolescent samples available for analysis.

Data are expressed as mean ± SEM, and differences were considered significant when *p* was ≤0.05.

Besides, correlation analysis was performed to evaluate the strength of the relationship between methylation of promoter fragments and mRNA expression.

Methylation rates are to be read as in the following example: a methylation rate of 0.2 means that 20% of DNA strands examined were methylated.

## Supplementary information

Supplemental Figure 1

Supplemental Table 1

Figure legends

## Data Availability

The datasets generated and analyzed during the study are available from the corresponding author on reasonable request.
