# Supplementary figures and images for: Methylation pattern and mRNA expression of synapse-relevant genes in the MAM model of schizophrenia in the time-course of adolescence
Source: Schizophrenia (Heidelb). 2022 Dec 8;8(1):110. doi: 10.1038/s41537-022-00319-8 (PMC9732294; doi:10.1038/s41537-022-00319-8)

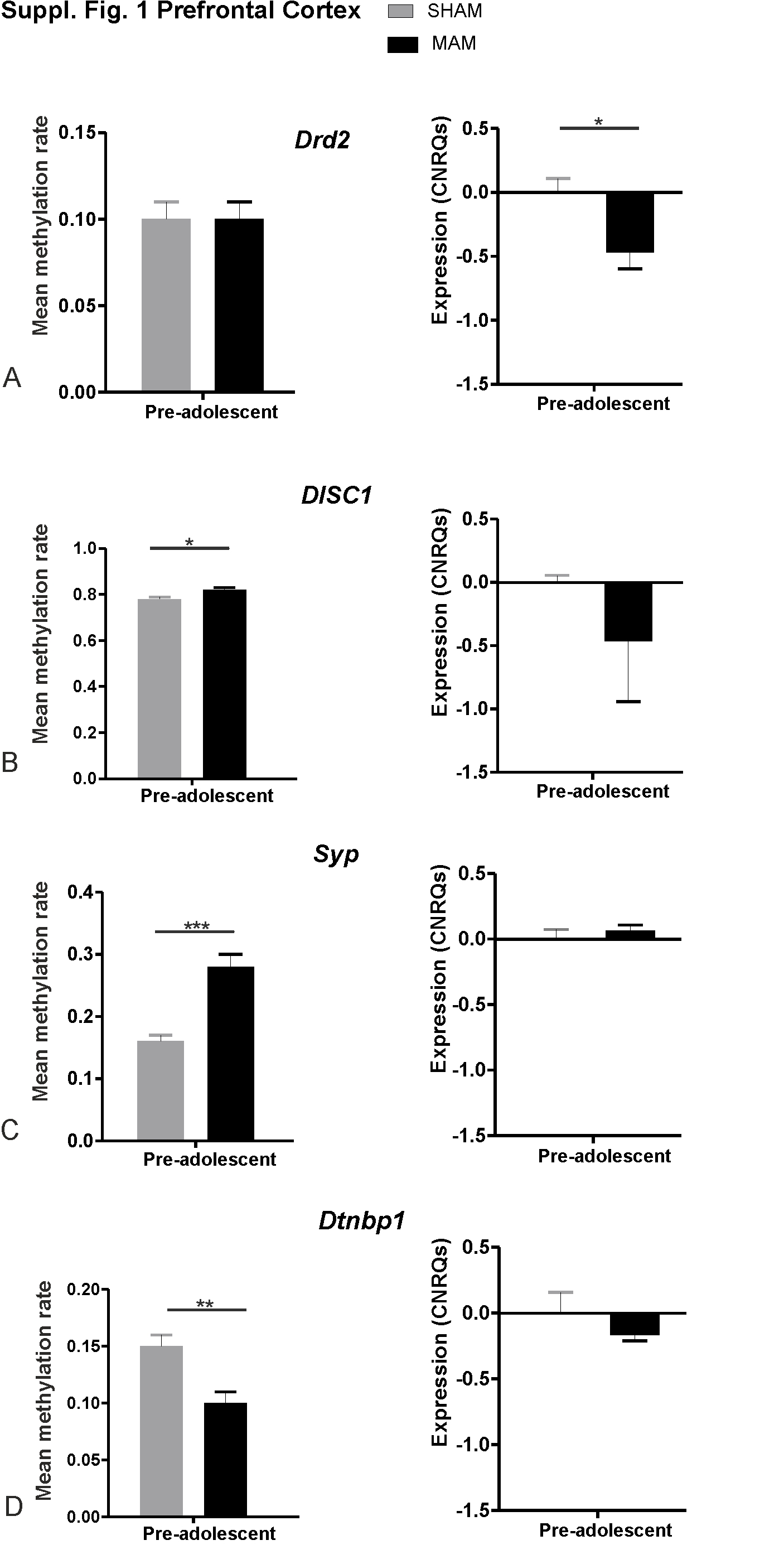

Supplement: Supplementary file 1 — Supplemental Figure 1 [file 41537_2022_319_MOESM1_ESM.jpg]
