# Supplementary material for: Methylation pattern and mRNA expression of synapse-relevant genes in the MAM model of schizophrenia in the time-course of adolescence
Source: Schizophrenia (Heidelb). 2022 Dec 8;8(1):110. doi: 10.1038/s41537-022-00319-8 (PMC9732294; doi:10.1038/s41537-022-00319-8)
Supplement: Supplementary file 2 — Supplemental Table 1 [file 41537_2022_319_MOESM2_ESM.docx]

| **Estimates of Fixed Effects** | | | | | | | | |
| --- | --- | --- | --- | --- | --- | --- | --- | --- |
| **Area** | **Substance** | **Puberty** |  | **Estimate** | **Std. Error** | **Sig.** | **95% Confidence Interval** | |
|  |  |  |  |  |  |  | **Lower Bound** | **Upper Bound** |
| **Prefrontal cortex (*Drd2*)** | **Sham** | Pre-adolescent | Methylation | **1.117** | 0.266 | **0.000** | 0.594 | 1.641 |
|  | **MAM** | Pre-adolescent | Methylation | **-0.260** | 0.110 | **0.019** | -0.477 | -0.043 |
| **Prefrontal cortex (*Disc1*)** | **Sham** | Pre-adolescent | Methylation | 0.629 | 0.499 | 0.210 | -0.357 | 1.616 |
|  | **MAM** | Pre-adolescent | Methylation | 0.121 | 0.0685 | 0.079 | -0.014 | 0.257 |
| **Prefrontal cortex (*Syp*)** | **Sham** | Pre-adolescent | Methylation | **0.151** | 0.042 | **0.000** | 0.067 | 0.235 |
|  | **MAM** | Pre-adolescent | Methylation | 0.041 | 0.053 | 0.440 | -0.064 | 0.147 |
| **Prefrontal cortex (*Dtnbp1*)** | **Sham** | Pre-adolescent | Methylation | **0.085** | 0.031 | **0.007** | 0.023 | 0.147 |
|  | **MAM** | Pre-adolescent | Methylation | **-0.902** | 0.140 | **0.000** | -1.178 | -0.626 |
| **Dependent Variable: Normalized_relative_quantities** | | | | | | | | |

Supplemental Table 1: Fixed effects calculated with SPSS for expression and methylation correlation estimates in the prefrontal cortex. Significant findings are presented in bold print. Of those, negative correlation estimates are highlighted in grey.
