# Supplementary material for: Methylation pattern and mRNA expression of synapse-relevant genes in the MAM model of schizophrenia in the time-course of adolescence
Source: Schizophrenia (Heidelb). 2022 Dec 8;8(1):110. doi: 10.1038/s41537-022-00319-8 (PMC9732294; doi:10.1038/s41537-022-00319-8)
Supplement: Supplementary file 3 — Figure legends [file 41537_2022_319_MOESM3_ESM.docx]

**Supplemental Figure 1**

Mean Methylation Rates and Expression levels (CNRQs, calibrated normalized relative quantities) of MAM and Sham pre-adolescent groups for **A** *Drd2*, **B** *DISC 1*, **C** *Syp and* **D** *Dtnp1* in PFC*.* Significant differences between two groups are indicated by a line with asterisks on top. The number of asterisks indicates the level of significance: *: p<0.05, **: p<0.01; ***: p<0.001. MAM: animals treated with Methylazoxymethanol-Acetate, Sham: animals only treated with vehicle. *Drd2*: dopamine receptor 22, *DISC1*: disrupted in schizophrenia 1, *Syp*: synaptophysin, *Dtnbp1*: dysbindin. For the analysis 7 pre-adolescent Sham, 6 pre-adolescent MAM, 7 post-adolescent Sham and 6 post-adolescent MAM-animals have been included.
